# Genome-wide CRISPR screen identifies ACSL3 as a regulator of lipotoxicity and progression of MASLD

**DOI:** 10.1097/HC9.0000000000000884

**Published:** 2026-01-21

**Authors:** Yuta Myojin, Takahiro Kodama, Ryo Takahashi, Hideharu Nagasawa, Yasuteru Kondo, Kosuke Yusa, Tomomi Yoshida-Hashidate, Hideo Shindou, Kunimaro Furuta, Kazuhiro Murai, Yoshinobu Saito, Hayato Hikita, Tetsuo Takehara

**Affiliations:** 1 Department of Gastroenterology and Hepatology, the University of Osaka, Graduate School of Medicine, Suita, Japan; 2 Department of Hepatology, Sendai Kousei Hospital, Sendai, Japan; 3 Department of Hepatology, Sendai Tokushukai Hospital, Sendai, Japan; 4 Department of Stem Cell Genetics, Institute for Life and Medical Sciences, Kyoto University, Kyoto, Japan; 5 Department of Lipid Life Science, National Institute of Global Health and Medicine, Japan Institute for Health Security, Tokyo, Japan; 6 Department of Medical Lipid Science, Graduate School of Medicine, The University of Tokyo, Tokyo, Japan; 7 Department of Gastroenterology and Hepatology, Ikeda Municipal Hospital, Osaka, Japan; 8 Department of Gastroenterology and Hepatology, Kansai Rosai Hospital, Amagasaki, Japan

**Keywords:** endoplasmic reticulum stress, hepatocyte apoptosis, lipid droplets, metabolic dysfunction–associated steatohepatitis, single-cell transcriptomics

## Abstract

**Background:**

Metabolic dysfunction–associated steatotic liver disease (MASLD) and its progressive form, metabolic dysfunction–associated steatohepatitis, are highly prevalent and lack effective pharmacotherapies. Hepatocellular lipotoxicity—driven by the accumulation of saturated fatty acids (eg, palmitate)—promotes disease progression; however, the determinants of hepatocyte susceptibility remain incompletely defined.

**Methods:**

We performed a genome-wide CRISPR-Cas9 loss-of-function screening to identify the regulators of palmitate-induced lipotoxicity. The top candidates were validated using genetic perturbation and pharmacological inhibition. Lipid handling, endoplasmic reticulum/oxidative stress, apoptosis, and lipogenic transcriptional programs were also quantified. Human MASLD liver tissues were analyzed for ACSL3 expression in relation to histology and aminotransferases. Single-cell and spatial transcriptomics were used to localize ACSL3 expression and the associated pathway signatures in metabolic dysfunction–associated steatohepatitis.

**Results:**

The screen recovered established mediators (CASPASE-8, AGPAT9, RNF213) and identified ACSL3 as a novel determinant of hepatocyte survival under lipotoxic stress. Genetic deletion or pharmacological inhibition of ACSL3 renders hepatocytes resistant to palmitate-induced apoptosis and endoplasmic reticulum stress, accompanied by reduced lipid-droplet accumulation, decreased incorporation of saturated fatty acids into neutral lipids and phospholipids, and blunted induction of lipogenic programs. In human MASLD, hepatic ACSL3 expression positively correlated with histological severity and aminotransferase levels. Single-cell transcriptomics localized ACSL3 predominantly to hepatocytes in advanced metabolic dysfunction–associated steatohepatitis displaying oxidative and endoplasmic reticulum stress signatures, whereas spatial transcriptomics showed ACSL3-high hepatocyte regions enriched for apoptotic and inflammatory pathways and spatially coupled to macrophage-rich and plasma cell–rich niches.

**Conclusions:**

ACSL3 is a central regulator of lipotoxic hepatocyte injury and MASLD progression, mechanistically linking lipid-droplet biogenesis to apoptosis and inflammatory niche formation. These data suggest that ACSL3 is a promising therapeutic target and support further translational studies to evaluate ACSL3 modulation in steatotic liver disease.

## Graphical abstract

**Figure:**
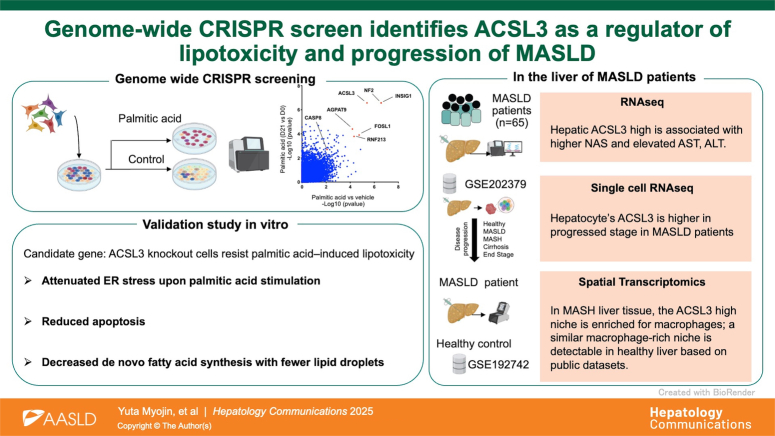

## INTRODUCTION

Metabolic dysfunction–associated steatotic liver disease (MASLD), formerly referred to as NAFLD, is one of the most prevalent chronic liver disorders worldwide and is tightly linked to obesity, type 2 diabetes, and dyslipidemia.^1^^,^^2^ Recent epidemiological studies have estimated that MASLD affects nearly 30% of the global population, with 3%–5% progressing to metabolic dysfunction–associated steatohepatitis (MASH), which carries a high risk of cirrhosis, liver failure, and HCC.^1^^,^^2^ Despite the urgent need for effective therapeutic strategies, the development of pharmacological treatments for MASH is proven to be difficult. Numerous clinical trials targeting diverse pathophysiological aspects of NAFLD Activity Score (NAS), such as inflammation, fibrosis, and lipid metabolism, have yielded limited or negative results for a while.^3^ Recently, the US Food and Drug Administration (FDA) approved 2 pharmacological agents for MASH: a thyroid hormone receptor-β agonist and a glucagon-like peptide-1 receptor agonist, marking a significant milestone.^4^^,^^5^ However, the modest efficacy of currently approved and investigational agents underscores the need for continued efforts to identify novel therapeutic targets and to develop more effective interventions.

A critical feature of MASLD progression is hepatocellular injury triggered by lipid overload, particularly the accumulation of saturated fatty acids such as palmitate.^6^ This phenomenon, termed lipotoxicity, promotes hepatocyte apoptosis, oxidative and endoplasmic reticulum (ER) stress, and inflammatory cell recruitment, ultimately driving the transition from simple steatosis to MASH and cirrhosis.^7^ Mechanistically, free fatty acids are released into the peripheral circulation through the activity of hormone-sensitive lipase in adipose tissue, whereas elevated hyperglycemia enhances de novo lipogenesis in hepatocytes. These disturbances impair mitochondrial function, reduce β-oxidation, and limit triglyceride export, resulting in intracellular lipid accumulation and cellular stress.^8^ Although these processes are recognized as key contributors to MASLD progression, the precise molecular mechanisms through which intracellular fatty acid overload triggers hepatocyte death remain unclear. Elucidating these pathways and identifying novel regulators within them are therefore critical for the development of effective therapeutic strategies.

High-throughput genetic screening technologies, such as a pooled CRISPR library screening, provide powerful tools to systematically investigate gene function and uncover previously unrecognized drivers of disease.^9^^–^^12^ In this study, we employed a genome-wide CRISPR-Cas9 knockout (KO) screen to systematically identify the genes that regulate hepatocyte survival under palmitic acid (PA)–induced lipotoxic stress. We validated the top candidates using KO and knockdown models in liver cells and primary hepatocytes, followed by multiomics analyses including lipid profiling, transcriptomics, and spatial transcriptomics of human liver tissue. Our findings revealed that acyl-CoA synthetase long-chain family member 3 (ACSL3) is a critical mediator of lipotoxic hepatocyte injury, linking lipid droplet biogenesis and lipogenic signaling to ER stress, apoptosis, and inflammatory niche formation in MASLD.

## METHODS

### Human samples and ethics

This was a retrospective cohort study that enrolled a total of 65 biopsy-proven MASLD patients diagnosed at 3 Japanese hospitals between 2014 and 2020. These patients satisfied the following criteria: (1) presence of fatty liver by liver imaging test (eg, hepatorenal contrast by ultrasonography), (2) absence of chronic liver disease other than MASLD (eg, viral hepatitis or autoimmune hepatitis), (3) alcohol consumption of <20 g/d, and (4) presence of at least one cardiometabolic risk factor including obesity, hyperglycemia or type 2 diabetes mellitus, hypertension, hypertriglyceridemia, low HDL-cholesterol levels, or increased waist circumference. Liver biopsy specimens were snap-frozen and stored at −80°C. All patients provided written informed consent, and the study design was consistent with the principles of the Declaration of Helsinki and Istanbul. The study protocol using patient tissues was approved by the Institutional Review Board Committees at Osaka University Hospital (Institutional Review Board No. 17097 and 19551).

### Cell lines and culture conditions

The human HCC cell line HLF^13^ was cultured in DMEM (high glucose, Sigma-Aldrich) supplemented with 10% fetal bovine serum (ThermoFisher Scientific) and 1% antibiotics (ThermoFisher Scientific) at 37 °C in a 5% CO_2_ humidified incubator. For lipotoxic stress experiments, PA (Sigma-Aldrich) was conjugated to fatty acid–free BSA (Sigma-Aldrich) at a 6:1 molar ratio and used at 200 μM unless otherwise indicated. Primary human hepatocytes were isolated from chimeric mice with >50% human liver replacement using a previously established 2-step collagenase–pronase perfusion method. The mice were housed at the Osaka University animal facility, in accordance with the ARRIVE statement and the guidelines provided by the National Research Council’s Guide for the Care and Use of Laboratory Animals.^14^

### Generation of Cas9-expressing cells and genome-wide CRISPR screen

HLF cells were transduced with a lentiviral vector encoding S. pyogenes Cas9 (lentiCas9-Blast, Addgene #52962) and selected with blasticidin for 14 days. Cas9 expression was confirmed by western blot in the previous paper.^13^ For the genome-wide KO screen, cells were transfected with lentivirus including whole genome-wide human single-guide RNA (sgRNA) library^10^ at 1.5 MOI to ensure single sgRNA integration per cell and selected with puromycin (1 μg/mL) for 7 days. A total of ~1×10^8^ cells were cultured under (1) control conditions or (2) 200 μM PA treatment for 3 weeks. Genomic DNA was extracted using the QIAamp DNA Blood Maxi Kit (QIAGEN), and sgRNA sequences were PCR-amplified and subjected to next-generation sequencing using Illumina platforms. Read counts were normalized and analyzed with MAGeCK to identify enriched or depleted sgRNAs.^9^^,^^10^^,^^12^

### CRISPR KO and RNA interference

CRISPR-Cas9–mediated KO of ACSL3 and INSIG1 was performed using individual sgRNAs cloned into lentiGuide-Puro vectors as we described.^14^ The reference sequences for ACSL3 were #1 CAACAGTGATGATGTGCCGC, #2 CGAGTGGATGATAGCTGCAC, and #3 CTCACAGAAGATGGCGATGT. The reference sequences for INSIG1 were #1 CAGCCCCTACCCCAACACC, #2 GTCACTCTCTTCCCCGAGG, and #3 TCAGCGTCCGGGGCACCGT. Transduced cells were selected using puromycin, and KO efficiency was validated by quantitative PCR and western blotting. For knockdown in primary human hepatocytes, cells were transfected with small interfering RNA targeting ACSL3 (s4998, s4999, Thermo Fisher) using Lipofectamine RNAiMAX (Thermo Fisher).

### Cell viability and apoptosis assays

Cell viability after PA treatment was assessed using the IncuCyte S3 live-cell analysis system (Sartorius). Apoptosis was evaluated using Annexin V–FITC/7-AAD staining (BD Pharmingen) followed by flow cytometry (BD Canto II). The FCS files were analyzed using FlowJo software (v10, Becton, Dickinson & Company).

### Lipid staining and quantification

Cells were fixed with 4% paraformaldehyde and stained with BODIPY 493/503 (Thermo Fisher Scientific) to visualize the lipid droplets. Images were acquired using fluorescence microscope (CQ-1 laser scanning confocal microscope, Yokogawa Electric Corporation).

### Quantification of fatty acids

Cells were treated with 200 μM PA for 6, 12, or 24 hours, washed in PBS, and harvested for lipid extraction using the Bligh and Dyer method.^15^ Fatty acids fractionation and measurements were performed as described.^16^ Briefly, the extracted samples were separated into the neutral lipid, the free fatty acid, and the phospholipid (PL) fractions using a solid phase column (InertSep NH2-cartridge, GL Science). Then, the unseparated sample and the 3 separated samples were spiked with 1 µg tricosanoic acid (C23:0) as an internal control and derivatized to fatty acid methyl esters using the Fatty Acid Methylation Kit (Nacalai Tesque). These derivatized samples were analyzed using gas chromatography-hydrogen flame ionization detection (GC-FID)(GC2010Plus, Shimadzu). PA concentration was determined based on internal standards.

### Gene expression analysis

RNA was isolated using the RNeasy Mini Kit (QIAGEN). Quantitative PCR was performed using the Thunderbird quantitative PCR Master Mix (Toyobo) and TaqMan probes (Applied Biosystems) on a QuantStudio 6 Flex system. The gene expression was normalized to that of β-actin. Primers used in the TaqMan assays (ThermoFisher Scientific) for ACSL3 (Hs00244853_m1), *CPT1A (*Hs00912671_m1), *CPT2 (*Hs00988962_m1), *ACOX1 (*Hs01074241_m1), *SCD (*Hs01682761_m1), ChREBP*(MLXIPL;* Hs00975714_m1), SREBP1*(SREBF1;* Hs02561944_s1), *PPARγ* (Hs01115513_m1), and *LXRα* (Nr1h3; *Hs00172885_m1*) were also used.

### Bulk RNA sequencing of liver biopsy tissue

Snap-frozen tissues from liver biopsies of 64 patients with MASLD at Sendai Kosei Hospital between December 2016 and August 2018 were used. Total RNA was extracted using the RNeasy kit (QIAGEN) according to the manufacturer’s protocol as described.^17^ RNA integrity was assessed, and only samples with an RNA integrity number >5 were advanced to RNA-seq. Library preparation was performed in the Genomics Core (Institute of Microbiology) using the TruSeq Stranded mRNA Sample Prep Kit (Illumina) on an Apollo Library Prep System (Takara). Sequencing was carried out on an Illumina HiSeq. 3000 in 75-bp single-end mode. Sequenced reads were aligned to the human reference genome (hg19) using TopHat v2.1.1, and gene-level expression (fragments per kilobase of transcript per million mapped reads) was quantified with Cuffnorm v2.2.1 (Cufflinks suite). The NAS of all biopsy hematoxylin and eosin (H&E) staining was assessed by an experienced pathologist at the Sendai Kosei Hospital. The study protocol using patient tissues was approved by the Institutional Review Board Committees at the University of Osaka Hospital (Institutional Review Board No. 17097). We uploaded these sequencing data to Zenode under the identification number 10.5281/zenodo.17062380.

### Single-cell and spatial transcriptomic analyses

Publicly available single-cell RNA-seq data from patients with MASLD were downloaded from GSE202379^18^ and spatial transcriptome data from GSE192742.^19^ Single-cell RNA-seq data in GSE192742 were used for reference in robust cell type decomposition analysis. The original cell annotation was used for the analysis, and healthy liver data were used for the annotation of our Visium and public Visium data from GSE 192742. Seurat (v4.3.0) and AU cells were used for single-cell RNA-seq analysis. For the pathway analysis, HALLMARK_APOPTOSIS, HALLMARK_REACTIVE_OXYGEN_ SPECIES, HALLMARK_GLYCOLYSIS, WP_OXIDATIVE_STRESS_RESPONSE, GOBP_ RESPONSE_TO_ENDOPLASMIC_RETICULUM_STRESS were used.

Spatial transcriptomics was performed on FFPE liver biopsy sections from a pathologically diagnosed MASH patient using the 10x Genomics Visium platform according to the manufacture’s protocol. The data were processed using Space Ranger. Data from 10x Visium were imported into Seurat as a Spatial assay with raw counts retained and mirrored as a SingleCellExperiment. The single-cell RNA sequencing reference from GSE192742 was read in 10x format and paired with the metadata file annot_humanAll.csv using the column annot as the cell type label after cleaning label strings and harmonizing barcodes by removing trailing hyphen one. Visium counts were log normalized using Seurat LogNormalize with a scale factor of thousand or scuttle logNormCounts and the column order was aligned to Seurat barcodes such that downstream analyses used the same population. ACSL3 classifications were then defined directly from this log normalized matrix as follows: among spots with ACSL3 expression the top 30% by ACSL3 level were labeled ACSL3 high and the bottom 30% were labeled ACSL3 low. To determine high and low ACSL3 expression, we used the upper 30% for high, the lower 30% for low, and defined the remainder as middle. We also tested whether the trends changed when using thresholds of 20% or 50%, and the trends did not change. Cell-type deconvolution was performed using robust cell type decomposition^20^ (spacexr) with a matched single-cell reference and Visium spot counts. Pathway activity for each spot was computed by gene set variation analysis^21^ using Hallmark gene sets from msigdbr.^22^ We analyzed apoptosis, TNFA signaling via NFKB, TGF beta signaling, fatty acid metabolism, cholesterol homeostasis, oxidative phosphorylation, and ROS pathway, retaining only sets with at least ten genes that overlapped the expression matrix, and stored the resulting scores in the Seurat metadata. Differences between ACSL3 high and ACSL3 low were tested using 2-sided Wilcoxon rank sum tests and adjusted by the Benjamini Hochberg false discovery rate.

### Statistics and reproducibility

Unless stated otherwise, data are presented as the mean±SD. Two-group comparisons were performed using the Wilcoxon rank-sum tests or Fisher exact tests. Multiple testing was controlled using the Benjamini-Hochberg false discovery rate. For CRISPR screens, MAGeCK provides gene-level scores and false discovery rate. Volcano plots used thresholds |log_2_ FC| ≥0.25 and false discovery rate <0.05 unless specified. We did not develop a new code for analyzing the single-cell data. All statistical analyses were performed using Prism (GraphPad, v8.4.3) or RStudio (v2024.12.0+467), using 2-sided tests with Benjamini-Hochberg correction where applicable.

## RESULTS

### Genome-wide CRISPR-Cas9 screen identifies mediators of lipotoxic stress

**FIGURE 1:**
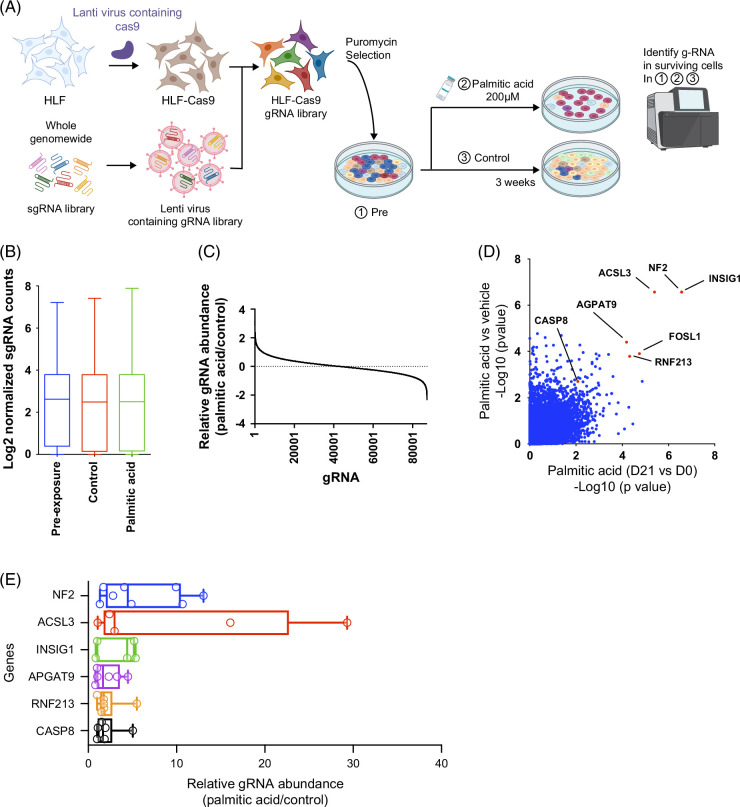
Genome-wide CRISPR-Cas9 screen identifies mediators of lipotoxic stress. (A) Schematic of the screening workflow in HLF cells stably expressing Cas9 and transduced with a genome-wide lentiviral sgRNA library; cultures were exposed for 3 weeks to 200 μM palmitic acid (PA) or control medium. (B) Bar plot of log2-normalized sgRNA counts for each group showing comparable library representation. (C) Distribution of sgRNA enrichment in PA versus control. (D) Gene-level scatter plot of −log10 *p* values; *x*-axis, PA versus vehicle; *y*-axis, day 14 versus day 0. (E) Log2 fold changes of individual sgRNAs in PA-treated versus control cells. Abbreviations: ACSL3, acyl-CoA synthetase long-chain family member 3; HLF, human liver cancer cell line; sgRNA, single-guide RNA.

To uncover the genetic determinants of lipotoxic stress, we conducted a genome-wide CRISPR-Cas9 KO screen of HLF liver cancer cells. As shown in Figure 1A, Cas9-expressing HLF cells were transduced with a lentiviral sgRNA library covering the human genome. Based on Supplemental Figure S1A, http://links.lww.com/HC9/C217, we selected 200 μM PA to induce lipotoxicity as it induced clear apoptosis while still permitting modest net cell expansion over 72 hours. After puromycin selection, cells were cultured for 3 weeks under either (1) 200 µM PA to induce lipotoxic stress or (2) vehicle control conditions. Genomic DNA was extracted, and sgRNA abundance was quantified by targeted deep sequencing. Library coverage was well-maintained across the pre-exposure, control, and treatment groups (Figure 1B). Comparative analysis revealed a marked shift in sgRNA distribution in PA-treated cells relative to that in the controls (Figure 1C). Notably, sgRNAs targeting *insulin-induced gene 1* (*INSIG1*), *neurofibromin 2* (*NF2*), and *acyl-CoA synthetase long-chain family member 3* (*ACSL3*) were significantly enriched in PA-treated cells compared to both control and pre-exposure conditions (Figures 1D–E; Supplemental Table S1, http://links.lww.com/HC9/C218). In addition, the screen recovered sgRNAs targeting *caspase8* (*CASP8*), *1-acylglycerol-3-phosphate O-acyltransferase 9* (*AGPAT9*: also known as *GPAT3*) and *ring finger protein 213* (*RNF213*), which have been previously implicated in regulating lipotoxicity and hepatocyte survival,^23^^,^^24^ thereby validating the robustness of our approach (Figures 1D, E). Collectively, these results demonstrate that our genome-wide CRISPR-Cas9 screening strategy successfully identified both established and novel mediators of lipotoxic stress, providing a valuable resource for dissecting the molecular basis of hepatocyte injury.

### ACSL3 loss confers resistance to PA-induced cell death

**FIGURE 2:**
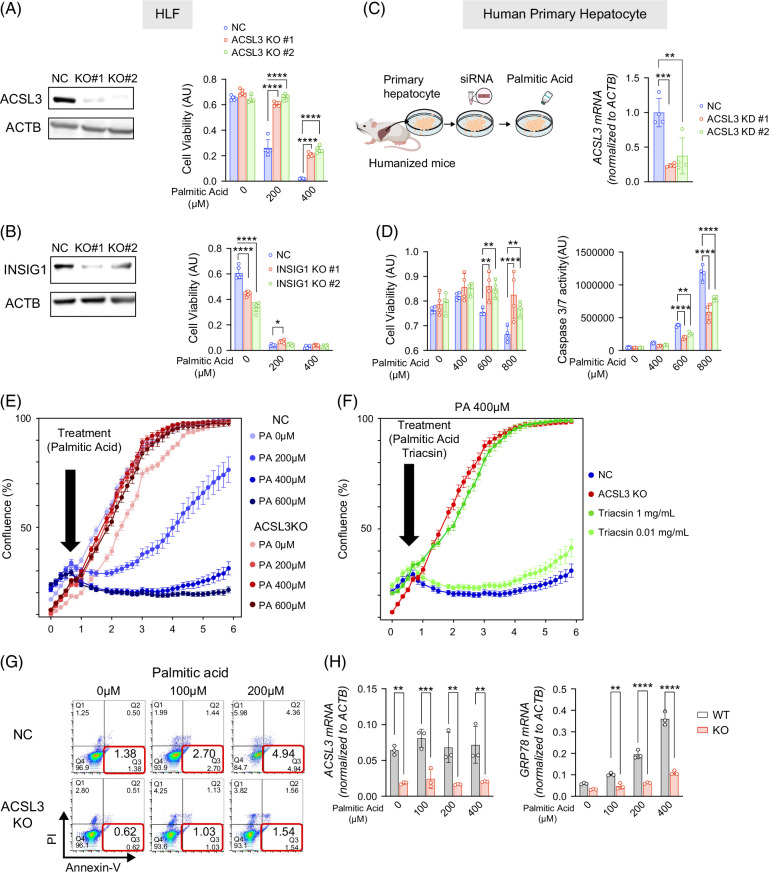
ACSL3 loss confers resistance to palmitic acid–induced cell death. (A) Left, ACSL3 protein by western blot; right, viability of ACSL3-KO and NC HLF cells after 72 hours PA. (B) Left, INSIG1 protein by western blot; right, viability of INSIG1-KO and NC HLF cells after 72 hours PA. (C, D) Primary human hepatocytes (isolated from humanized mice) transfected with ACSL3 siRNA; ACSL3 mRNA by RT-qPCR normalized to ACTB (C), and PA-induced changes in viability and caspase-3/7 activity (D). (E) Real-time imaging (IncuCyte) of confluence in ACSL3-KO and NC HLF cells during day 1–6 PA exposure. (F) Viability of HLF cells treated with PA with or without the pan-ACSL inhibitor triacsin. (G) Flow-cytometric quantification of Annexin V and propidium iodide-positive cells after 24 hours PA in ACSL3-KO versus NC HLF cells. (H) ACSL3 and GRP78 mRNA by RT-qPCR normalized to ACTB following PA exposure. Abbreviations: ACSL3, acyl-CoA synthetase long-chain family member 3; KO, knockout; NC, negative control; PA, palmitic acid; siRNA, small interfering RNA; WT, wild type.

Given that *NF2* is a well-established tumor suppressor gene,^25^ we focused on subsequent analyses of *INSIG1* and *ACSL3*. Each gene was deleted in HLF cells using CRISPR, and the response to PA was assessed. As shown in Figure 2A, only ACSL3 deletion conferred resistance to PA-induced cytotoxicity, whereas INSIG1 deletion resulted in sensitivity comparable to that of the negative control cells (Figure 2B). This may reflect functional redundancy between INSIG1 and INSIG2^26^ and adaptive feedback within the SREBP-INSIG axis.^27^ To determine whether ACSL3 also modulates lipotoxicity induced by fatty acids other than PA, we performed dose-response assays with myristic acid, stearic acid, and oleic acid. ACSL3 KO reduced myristic acid–induced cytotoxicity, similar to the effect observed with palmitate, whereas stearic acid and oleic acid elicited little toxicity and showed no detectable difference between control and ACSL3-KO cells (Supplemental Figure S1B, http://links.lww.com/HC9/C217). To validate these findings in a more physiological context, ACSL3 expression was silenced using small interfering RNA in primary human hepatocytes. ACSL3 inhibition attenuated the PA-induced loss of viability and reduced caspase-3/7 activity, indicating enhanced resistance to cell death (Figures 2C, D). This protective effect was further supported by real-time imaging using the IncuCyte system, which showed increased survival of ACSL3-suppressed HLF cells (Figure 2E) and was recapitulated by pharmacological inhibition with the pan-ACSL inhibitor triacsin (Figure 2F). Annexin V/PI staining confirmed that PA exposure markedly increased apoptosis in parental cells but not in ACSL3 KO cells (Figure 2G). Moreover, ACSL3 KO cells failed to upregulate the ER stress marker GRP78 in PA treatment (Figure 2H). Collectively, these results established that ACSL3 is a critical determinant of hepatocyte susceptibility to lipotoxicity, linking its expression to PA-induced apoptosis and ER stress.

### ACSL3 loss reduces lipid droplet accumulation and intracellular fatty acid content

**FIGURE 3:**
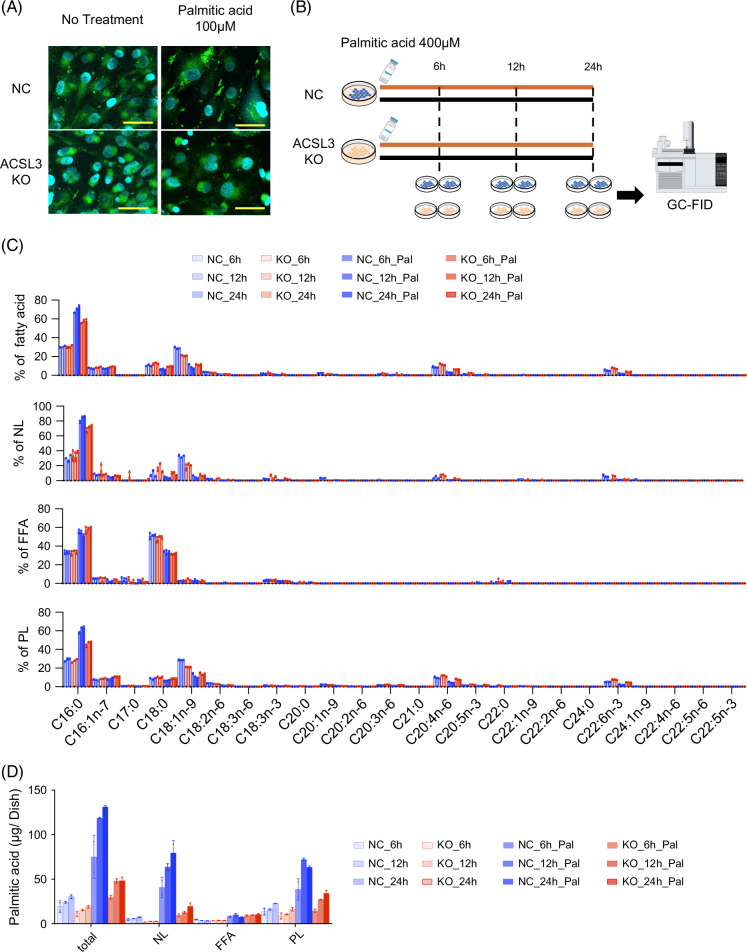
ACSL3 loss reduces lipid droplet accumulation and intracellular fatty acid content. (A) Representative lipid droplet staining in ACSL3-KO and NC HLF cells after PA exposure. (B) Timeline for the sample collection measured using GC-FID at 6, 12, and 24 hours post-PA. (C) Bar plots showing the proportion of each fatty acids containing unseparated fraction (total), and the separated fatty acid fractions in NL, FFA, and PL (n = 2 per group). (D) Bar plots showing absolute palmitate per dish for each condition. Abbreviations: ACSL3, acyl-CoA synthetase long-chain family member 3; FFA, free fatty acid; GC-FID, gas chromatography-hydrogen flame ionization detection; KO, knockout; NC, negative control; NL, neutral lipid; PL, phospholipids.

Because ACSL3 was associated with resistance to lipotoxicity, we investigated whether lipid droplet formation was altered in ACSL3 KO cells following PA exposure. As shown in Figure 3A, lipid droplet accumulation was markedly reduced in ACSL3 KO cells compared to controls, consistent with previous observation.^28^ Next, we quantified fatty acids using GC-FID to assess the intracellular fatty acid contents. Control and ACSL3 KO cells were treated with PA and collected at 6-, 12-, and 24-hours post-treatment (Figure 3B). These results revealed decreased levels of saturated fatty acids C16:0 (PA) in ACSL3 KO cells relative to controls following treatment with PA, particularly in neutral lipids and PL (Figure 3C). Absolute quantification of PA confirmed this reduction in ACSL3-deficient cells (Figure 3D).

### ACSL3 deficiency abrogates PA-induced activation of lipogenic programs without enhancing β-oxidation

**FIGURE 4:**
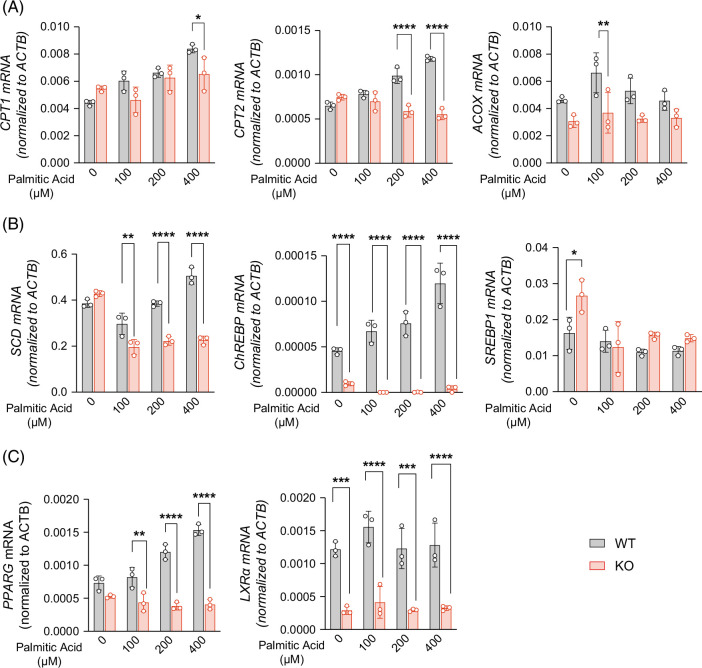
ACSL3 deficiency abrogates PA-induced activation of lipogenic programs without enhancing β-oxidation. (A–C) mRNA levels by RT-qPCR normalized to ACTB: CPT1, CPT2, and ACOX (A); SCD, ChREBP, and SREBP1 (B); PPARG and LXRA (C). Abbreviations: KO, knockout; WT, wild type.

To elucidate the mechanism underlying reduced lipid droplet accumulation, we examined the fatty acid metabolic pathways in ACSL3 KO and control cells. As shown in Figure 4A, β-oxidation markers (*CPT1*, *CPT2*, *ACOX*) were robustly induced by PA in wild-type cells, but were not further elevated in the ACSL3 KO cells, indicating that enhanced fatty acid catabolism is not the primary driver of the phenotype. In contrast, lipogenic gene expression was markedly impaired in the ACSL3-deficient cells. Although PA stimulation strongly upregulated *SCD* and *ChREBP* in wild-type cells, this induction was completely abolished in ACSL3 KO cells, where both genes remained significantly downregulated relative to the controls (Figure 4B). Moreover, the nuclear regulators *PPARγ* and *LXRα* were reduced at baseline in ACSL3 KO cells but were not induced by PA (Figure 4C). Together, these findings establish that ACSL3 is required for PA-induced activation of lipogenic pathways, and that its loss results in persistent suppression of lipid synthesis programs rather than compensatory upregulation of β-oxidation.

### Hepatic ACSL3 expression is elevated in MASLD and correlates with disease severity

**FIGURE 5:**
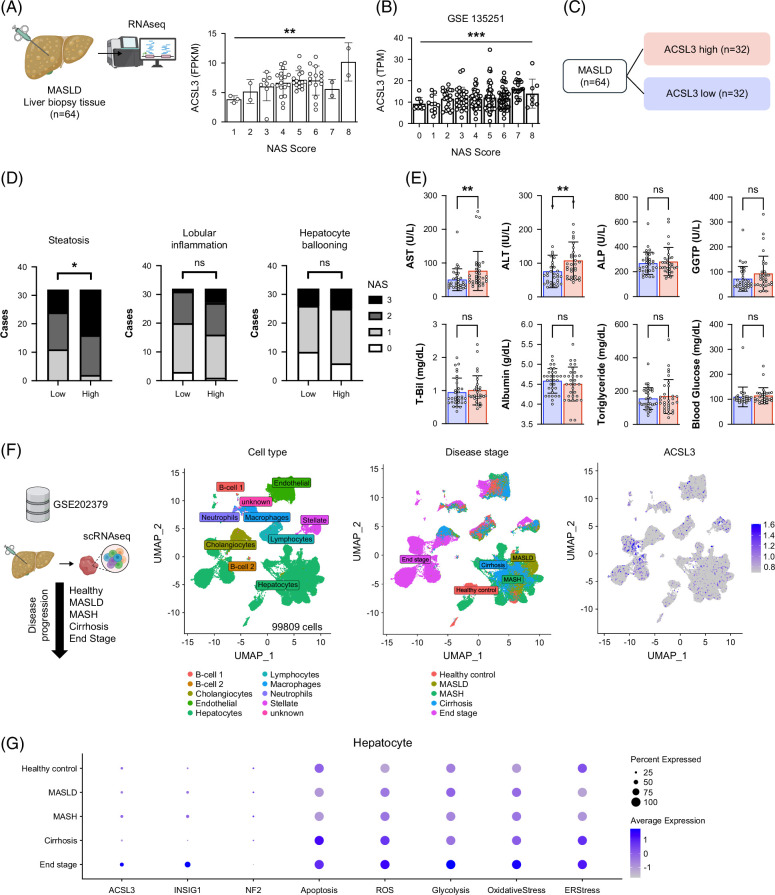
Hepatic ACSL3 expression is elevated in MASLD and correlates with disease severity. (A–E) Bulk RNA-seq of liver biopsies: hepatic ACSL3 versus NAFLD Activity Score (NAS) (A); hepatic ACSL3 versus NAFLD Activity Score (NAS) in GSE 135351 (B), NAS subscores by median split of hepatic ACSL3 (high versus low) (C–D); serum laboratory values (AST, ALT, ALP, GGT, total bilirubin, albumin, triglyceride, glucose) by ACSL3 group (E). (F, G) Public single-cell RNA-seq dataset GSE202379: UMAP colored by cell type, disease stage, and ACSL3 expression (F); dot plot of gene expression and pathway enrichment scores (G). Abbreviations: ACSL3, acyl-CoA synthetase long-chain family member 3; MASH, metabolic dysfunction–associated steatohepatitis; MASLD, metabolic dysfunction–associated steatotic liver disease; ROS, reactive oxygen species.

To assess the clinical relevance of our screening results, we evaluated ACSL3 expression in human liver tissues. Bulk RNA-seq analysis of liver biopsy specimens from patients with MASLD demonstrated that hepatic ACSL3 expression increased stepwise with higher NAS, indicating a positive association with histological severity (Figure 5A). To validate these results, we analyzed the different public available dataset (GSE135251)^29^ and the ACSL3 expression increased as NAS score increased (Figure 5B). Stratification by median expression further revealed that patients in the high-ACSL3 group exhibited higher NAS lipid subscores (Figure 5C, D) and significantly elevated serum AST and ALT levels compared to those in the low ACSL3 group (Figure 5E, Table 1). To further define the cellular context, we analyzed publicly available single-cell RNA-seq datasets. ACSL3 was most abundantly expressed in hepatocytes, particularly in advanced MASH tissues, which displayed transcriptional signatures of oxidative stress, reactive oxygen species (ROS), and ER stress (Figures 5F, G). Together, these findings establish that hepatocellular ACSL3 expression is upregulated in MASLD and is closely associated with both the histological and biochemical indices of disease severity.

**TABLE 1:** Clinical data of ACSL3-high and ACSL3-low group

		All (IQR) (n-64)	Missing#	ACSL3 low (IQR) (n=32)	ACSL3 high (IQR) (n-32)	*p* value (ACSL3 high vs. low)
Age	Years old	59.5 (47.3–66.8)	0	59.5 (48.3–66.8)	59.0 (45.8–65.0)	0.5819
Sex	Male/female	34/30	0	14/18	20/12	0.2101
Fibrosis	NL/CH/LC	2/60/2	0	2/29/1	0/31/1	0.3558
NAS_lipid	0/1/2/3	0/13/27/24	0	0/11/13/8	0/2/14/16	**0.0115**
NAS_inflammation	0/1/2/3	4/32/22/6	0	3/17/11/1	1/15/11/5	0.2839
NAS_ballooning	0/1/2	16/35/13	0	10/16/6	6/19/7	0.5132
Height	cm	163.6 (154.0–168.1)	0	161.6 (152.7–166.8)	165.7 (154.1–171.5)	0.0954
Body weight	kg	74.3 (63.5–87.4)	0	71.3 (63.5–77.4)	80.5 (63.5–95.6)	**0.0403**
WBC	/µL	6400 (5500–7675)	0	6050 (5125–8025)	6500 (5825–7575)	0.3079
Platlet	×10^4^/µL	22.9 (17.7–27.1)	0	22.3 (16.3–27.1)	23.0 (18.6–27.5)	0.369
PT-INR		0.97 (0.94–1.01)	0	0.98 (0.94–1.04)	0.96 (0.92–0.99)	0.1014
AST	U/L	49.5 (35.3–71.5)	0	39.5 (31.3–61)	56.5 (43.5–88)	**0.0032**
ALT	U/L	78 (51.5–120.3)	0	63.5 (41.5–92.5)	96.5 (61–130)	**0.0035**
T-Bil	mg/dL	0.84 (0.67–1.11)	0	0.77 (0.64–1.19)	0.84 (0.79–1.11)	0.2597
GGTP	U/L	65.5 (45.3–100.8)	0	54.0 (40.5–99.5)	73 (49–100.8)	0.1625
ALP	U/L	242 (208–310)	0	251 (208–308)	242 (210–310)	0.9336
Albumin	g/dL	4.6 (4.2–4.8)	0	4.6 (4.3–4.8)	4.5 (4.2–4.8)	0.4109
Toriglyceride	mg/dL	132 (106–204)	0	127 (113–204)	138 (99–219)	0.9654
LDL-Chol	mg/dL	124 (98–154)	0	128 (117–159)	119 (93–151)	0.1158
Glucose	mg/dL	104 (94–120)	6	102 (94–110)	106 (95–125)	0.305
HbA1c	%	6.0 (5.6–6.4)	7	6.0 (5.6–6.4)	6.0 (5.6–6.6)	0.5012
DM	Yes/no	9/55	0	4/28	5/27	>0.9999
HL	Yes/no	19/45	0	5/27	14/18	**0.0272**
HT	Yes/no	24/40	0	10/22	14/18	0.439

Abbreviations: ACSL3, acyl-CoA synthetase long-chain family member 3; DM, diabetes mellitus; GGTP, gamma-glutamyl transpeptidase; HL, hyperlipidemia; HT,hypertension; NAS, NAFLD Activity Score; PT-INR, prothrombin time–international normalized ratio.

### Spatial transcriptomics links ACSL3-high hepatocytes to apoptotic signaling and an inflammatory niche

**FIGURE 6:**
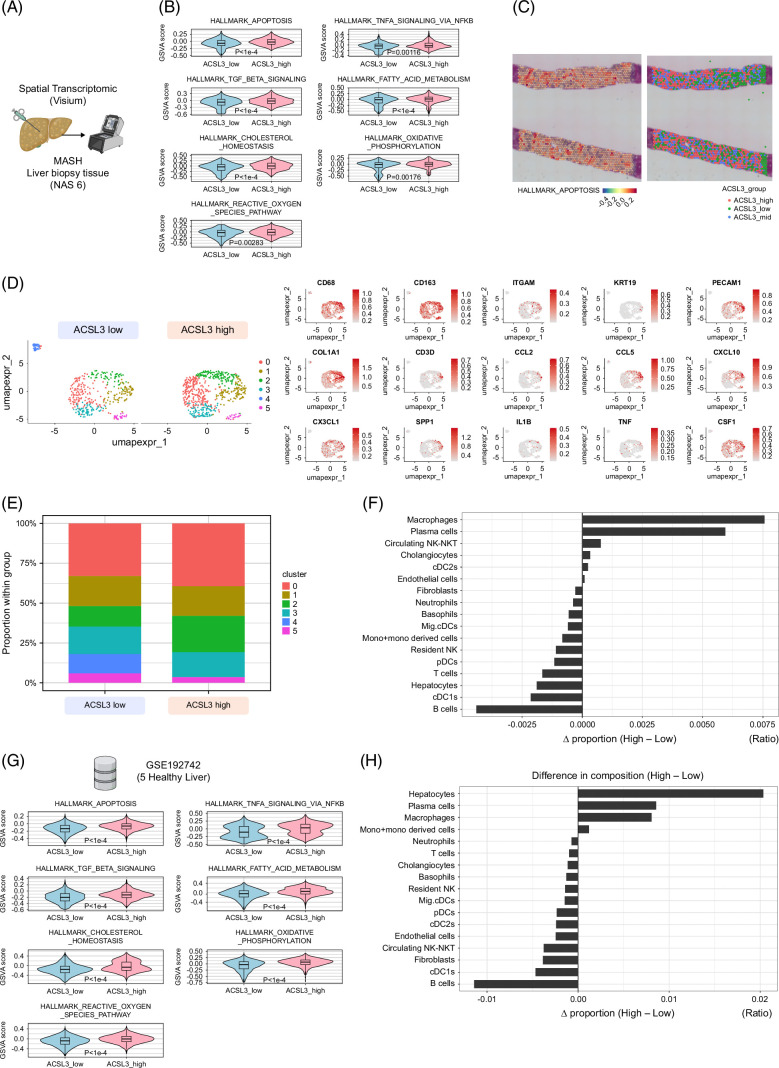
Spatial transcriptomics links ACSL3-high hepatocytes to apoptotic signaling and an inflammatory niche. (A) Visium spatial transcriptomics from a MASH biopsy; ACSL3-high regions defined by ranking spots by ACSL3 expression within the lesion and selecting the top 30th percentile. (B, C) Pathway scoring across spots using Hallmark gene sets (msigdbr) and GSVA; enrichment statistics as indicated, with multiple-testing correction where applicable. Representative spatial feature maps show the apoptosis score and ACSL3 high/low classification. (D) UMAP colored by Leiden clusters stratified by ACSL3 expression; marker-gene overlays shown at right. (E) Bar plot of cluster composition by ACSL3 group. (F) Cell-type deconvolution by RCTD showing inferred proportions across ACSL3-high versus ACSL3-low regions and the high-low difference. (G, H) Analysis of an independent public dataset (GSE192742) summarizing pathway scores and inferred cell-type composition across spots using the same ACSL3-based regional definition.

Next, we performed spatial transcriptomic profiling of a biopsy specimens from a patient with MASH. ACSL3-high regions, defined as the top 30th percentile of ACSL3-expressing spots, were subjected to pathway enrichment analysis and showed significant activation of hallmark apoptosis, TNF signaling via NF-κB, TGF-β signaling, lipid metabolism, and ROS pathways (Figures 6A–C). Cellular composition analysis with GSE192742^19^ as cell annotation reference, further revealed that cluster 2, enriched for CD68+ macrophages, was preferentially localized within ACSL3-high niches (Figure 6D–E), and this co-localization is consistent with prior studies linking lipotoxic hepatocyte stress to macrophage recruitment and phenotypic remodeling in MASH.^30^ To examine whether ACSL3 reflects hepatic zonation, we computed periportal (zone 1) and pericentral (zone 3) zonation scores from published signatures^31^^,^^32^ and mapped them onto the spatial transcriptomic profiles. Across biopsies, ACSL3-high regions did not consistently colocalize with either zone 1, zone 3 signatures, or lipid droplet distribution (Supplemental Figure S1C, http://links.lww.com/HC9/C217). Independent validation by cell-type deconvolution, using GSE192742 dataset,^19^ confirmed that ACSL3-high regions harbored increased infiltration of macrophages and plasma cells, consistent with a more inflammatory microenvironment (Figure 6F). Interestingly, similar immune cell enrichment patterns were also observed in the spatial transcriptomic data from healthy liver tissues (Figures 6G, H), suggesting that hepatocellular ACSL3 upregulation promotes lipid accumulation and cellular stress, thereby driving immune cell recruitment. Collectively, these results link ACSL3-high hepatocytes to apoptotic signaling establish an inflammatory niche, supporting a mechanistic role for ACSL3 in MASLD progression.

## DISCUSSION

In this study, we combined genome-wide CRISPR-Cas9 screening, functional validation, and multiomics analyses to identify ACSL3 as a critical determinant of hepatocyte susceptibility to lipotoxic stress. Our screen revealed that sgRNAs targeting ACSL3 were selectively enriched under PA exposure, and subsequent KO and knockdown studies confirmed that the loss of ACSL3 confers resistance to PA-induced apoptosis and ER stress. Mechanistically, ACSL3-deficient cells displayed impaired lipid droplet accumulation, reduced incorporation of saturated fatty acids into neutral lipids and PLs, and marked suppression of lipogenic transcriptional programs, including SCD, ChREBP, PPARγ, and LXRα. These findings highlight the essential role of ACSL3 in sustaining lipid biosynthesis and droplet formation, thereby linking lipid handling to the cell fate under lipotoxic conditions.

Beyond its pathological role, our findings provide insights into the physiological functions of ACSL3. Lipid droplets are recognized as metabolically active organelles that regulate fatty acid flux and cellular stress responses rather than inert storage depots. In MASLD, alterations in lipid droplets architecture and lipid droplts–organelle interactions, particularly with the ER, have been linked to ER stress, inflammation, and disease progression.^33^ Consistent with this concept, our data show that ACSL3 KO reduces LD formation in palmitate-loaded hepatoma cells and concomitantly attenuates ER stress markers and palmitate-induced cell death, indicating that ACSL3-dependent LD biogenesis under saturated fatty acid overload promotes, rather than protects from, lipotoxic stress. These findings support the notion that not only the quantity but also the quality and metabolic routing of LDs determine whether steatosis remains metabolically benign or is coupled to hepatocyte injury, and they position ACSL3 as a key regulator at the interface between LD dynamics and hepatocellular stress. Beyond its pathological role, our findings provide insights into the physiological functions of ACSL3. As a member of the acyl-CoA synthetase long-chain family, ACSL3 catalyzes the activation of long-chain fatty acids, channeling them into β-oxidation, glycerophospholipid synthesis, and membrane remodeling.^26^^,^^34^ Its localization to the ER and lipid droplets positions ACSL3 as a gatekeeper of hepatocellular lipid partitioning under normal conditions, thereby maintaining the metabolic balance.^35^^,^^36^ However, under nutrient excess and chronic palmitate exposure, this canonical activity becomes maladaptive; sustained ACSL3 function drives aberrant LD accumulation, perturbs ER homeostasis, and predisposes hepatocytes to oxidative and apoptotic stress. Thus, ACSL3 is protective for physiological lipid handling yet pathogenic in the setting of lipotoxic stress.

As a member of the acyl-CoA synthetase long-chain family, ACSL3 catalyzes the activation of long-chain fatty acids and channels them into mitochondrial β-oxidation, glycerophospholipid synthesis, and membrane remodeling. Increased delivery of long-chain acyl-CoAs into β-oxidation has been proposed to impose redox pressure on the electron transport chain, enhance mitochondrial superoxide and hydrogen peroxide formation, and thereby promote intrinsic, mitochondria-dependent apoptosis in hepatocytes.^37^^–^^39^ This framework is consistent with our observation that ACSL3 loss attenuates palmitate-induced caspase-3/7 activation and cell death, although mitochondrial respiration and ROS production were not directly assessed in this study.

Clinically, *ACSL3* expression was the highest in MASLD and scaled with histologic activity and aminotransferases, positioning *ACSL3* as a potential biomarker of disease activity. Single-cell transcriptomic data localized ACSL3 expression primarily in hepatocytes in advanced MASH, which simultaneously exhibited signatures of oxidative and ER stress signatures. Spatial transcriptomics extended these findings by demonstrating that ACSL3-high hepatocyte regions were enriched for apoptotic and proinflammatory pathways and spatially coupled with macrophage-rich and plasma cell–rich niches. This suggests that ACSL3 not only governs hepatocyte vulnerability to lipotoxicity but also shapes the inflammatory microenvironment that drives disease progression. This is consistent with established links between hepatocyte death signaling, macrophage recruitment, activation, and MASLD progression.^40^^,^^41^

This study had several limitations should be acknowledged. First, our functional validation was primarily performed in HLF liver cancer cells and primary human hepatocytes, which may not fully capture the complexity of the in vivo liver physiology. We recognize that cancer lines differ from normal hepatocytes in oncogenic signaling and metabolic wiring. We reduced this concern by validating key findings in primary hepatocytes, and interpreting screen hits through pathways conserved in hepatocytes. Second, although spatial transcriptomic data provide correlative evidence linking ACSL3-high hepatocytes to immune cell infiltration, causal interactions remain to be demonstrated in vivo. Third, although pharmacological inhibition recapitulates the genetic findings, the specificity of the available ACSL inhibitors remains limited. Future studies using hepatocyte-specific ACSL3 KO models and more selective inhibitors are essential to confirm the therapeutic potential of targeting ACSL3 in MASLD. Finally, our pooled CRISPR screen was designed with up to 8 sgRNAs per gene; however, after standard quality control at T0 and under selection with 200 µM PA, some guides were filtered out or dropped out biologically. As a result, gene-level summaries for a subset of genes are based on 5–6 detected sgRNAs. Unequal guide representation and occasional dropout can attenuate effect-size estimates and increase variance, potentially yielding false negatives. To mitigate this, we required minimum T0 counts and presence across replicates, summarized gene-level effects with MAGeCK.

In conclusion, we have identified ACSL3 as a central regulator of hepatocyte lipotoxicity and MASLD progression. ACSL3 integrates metabolic and stress pathways that shape the liver disease trajectory by coupling lipid droplet biogenesis with apoptosis and inflammatory niche formation. Our results not only advance the mechanistic understanding of lipotoxic liver injury but also indicate that ACSL3 as a promising target for therapeutic intervention in metabolic liver disease.

## Supplementary Material
