# Supplementary material for: Genome-wide CRISPR screen identifies ACSL3 as a regulator of lipotoxicity and progression of MASLD
Source: Hepatol Commun. 2026 Jan 21;10(2):e0884. doi: 10.1097/HC9.0000000000000884 (PMC12826143; doi:10.1097/HC9.0000000000000884)
Supplement: Supplementary file 1 [file hc9-10-e0884-s001.pdf]

Supplementary Figure legend

Supplementary Figure S1

A. Cell viability (left) and supernatant caspase-3/7 activity (right) of ACSL3-KO and negative control (NC) HLF cells after 24, 48, and 72 h of treatment with different concentrations of palmitic acid (PA).

B. Cell viability of ACSL3-KO and NC HLF cells after 72 h PA, SA, MA, or OA. Cell viability was normalized to the 0 μM treatment in each group (n=5, repeated twice).

C. Spatial plots of hepatic zonation based n GSEA using periportal (zone 1; CPS1, HAL, PCK1, ASS1, HAO1, ALDH6A1, ARG1, GLS2, SULT2A1) and pericentral (zone 3; GLUL, CYP2E1, CYP1A2, OAT, ADH1C, CYP2A6, CYP3A4, ALDH1A1, RHBG) gene sets, together with the spatial distribution of lipid droplet–containing areas and regions stratified by ACSL3 expression.

Figure s1

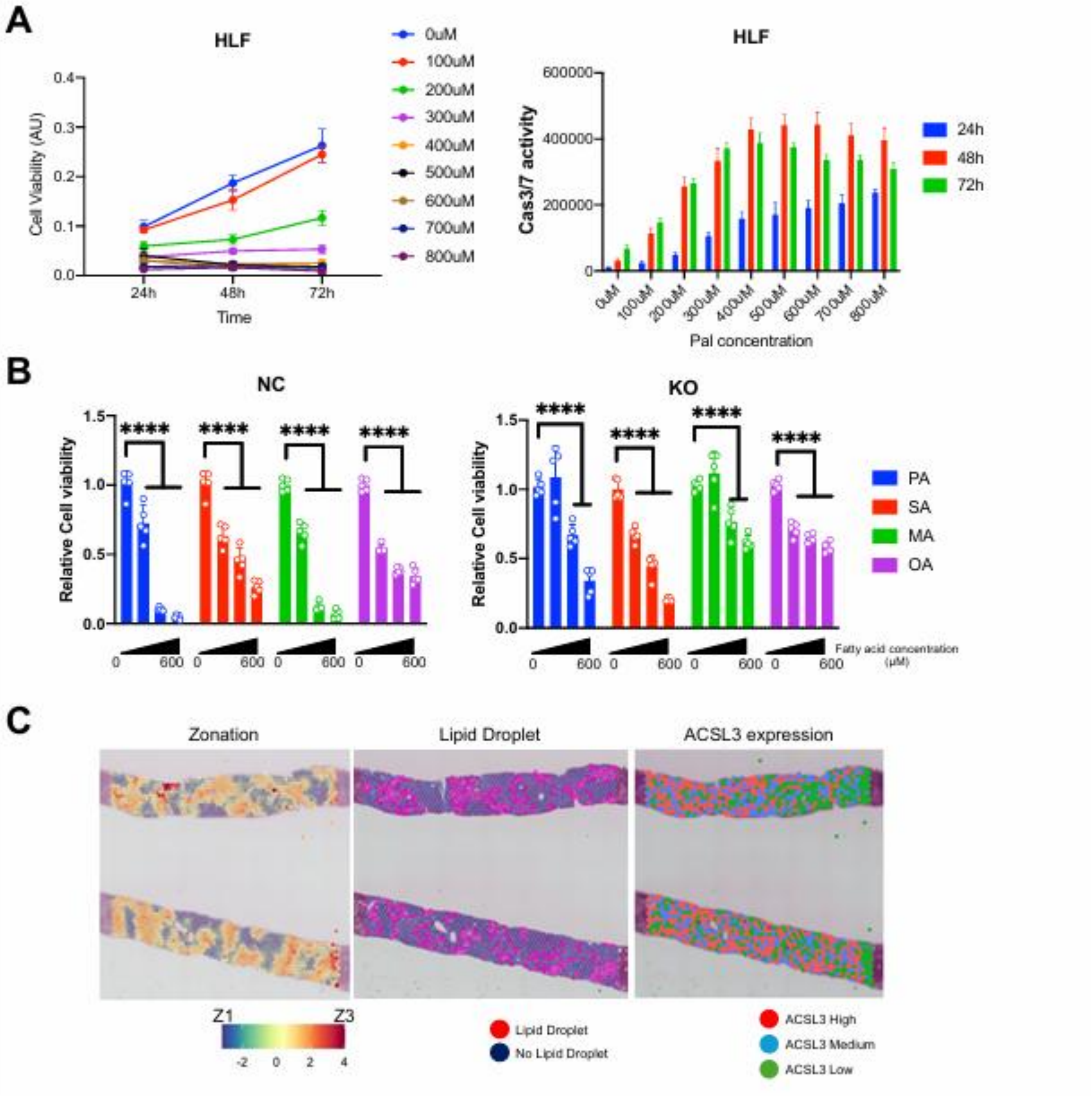
A
HLF
Cell Viability (AU)
0.4
0.3
0.2
0.1
0.0
24h
48h
72h
Time
0uM
100uM
200uM
300uM
400uM
500uM
600uM
700uM
800uM
HLF
Cas3/7 activity
600000
400000
200000
0
0uM
100uM
200uM
300uM
400uM
500uM
600uM
700uM
800uM
Pal concentration
24h
48h
72h
B
NC
KO
Relative Cell viability
1.5
1.0
0.5
0.0
****
0
600
Fatty acid concentration (μM)
PA
SA
MA
OA
C
Zonation
Lipid Droplet
ACSL3 expression
Z1
Z3
-2
0
2
4
Lipid Droplet
No Lipid Droplet
ACSL3 High
ACSL3 Medium
ACSL3 Low
